# Real-Time Binding Kinetics of Small Molecules to CA IX in Live Suspension Cells Using SPR Microscopy

**DOI:** 10.1021/acsmedchemlett.5c00555

**Published:** 2025-12-20

**Authors:** Miyuki A. Thirumurthy, Jesús Aguilar Díaz de león, Nguyen Ly

**Affiliations:** Biosensing Instrument Inc., Tempe, Arizona 85284, United States

**Keywords:** Carbonic anhydrase IX, sulfonamide inhibitors, SPRM, label-free detection, live-cell binding kinetics, membrane proteins

## Abstract

Membrane-associated carbonic anhydrase (CA IX) is overexpressed in multiple cancers, making it a compelling target for therapeutics, yet measuring small molecule binding is challenging outside its native environment. Surface Plasmon Resonance Microscopy (SPRM) enables label-free kinetic measurements on whole cells, revealing critical insights that are often missed by conventional assays that require receptor purification. Here, we pioneer the use of SPRM to study kinetic interactions of five sulfonamide-based small molecule inhibitors (Acetazolamide, Sulfanilamide Furosemide, Dansylamide, and 4-Carboxybenzenesulfonamide­(4-CBS)) with CA IX on live Ramos B suspension cells. SPRM measurements were in close agreement with the literature and demonstrated a low coefficient of variation (% CV) of 6.8%. Additionally, Sulfanilamide demonstrated a 16-fold stronger affinity in its native membrane-bound state than in its purified state. This pioneering study establishes SPRM for label-free kinetic measurements of small molecule interactions on live suspension cells *in vitro*.

## Graphical abstract

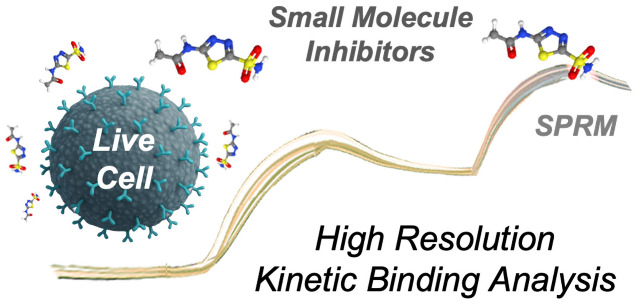

## Introduction

Carbonic anhydrases (CAs) constitute a family of zinc metalloenzymes that effectively catalyze the reversible hydration of carbon dioxide to bicarbonate. Out of the 15 different human CA isoforms, carbonic anhydrase IX (CA IX) has been of particular interest due to its restricted expression in normal tissues and widespread overexpression in malignant cells.^1^ Its tissue-restricted expression pattern, along with its role in the regulation of pH, makes CA IX a central regulator of survival of cancer cells and drug resistance.^2,3^ In addition, its membrane association on the extracellular side is a favorable aspect for drug targeting with minimal interference.^4^ Although CA IX has been extensively studied in solid tumors such as renal cell carcinoma and glioblastoma, its characterization in hematological malignancies remains historically scarce.^5,6^ Recent evidence has shown that CA IX is expressed in certain B-cell lymphomas, including Burkitt lymphoma, and it plays a vital role in metabolic adaptation, such as hypoxia and stress caused by a deficiency of nutrients.^7^ Given its surface location and tumor specificity, CA IX presents a unique opportunity for therapeutic intervention in lymphomas particularly through extracellularly acting drugs that avoid off-target cytoplasmic effects.^7,8^ However, isolating and purifying the full-length form of CA IX without losing its structural context is often very challenging.^9^ Most of the reported binding studies for CA IX use recombinant extracellular domains lacking membrane anchoring, which could misrepresent crucial aspects of binding kinetics and inhibitor access.^10^ To probe CA IX function and its interactions with small-molecule inhibitors in a physiologically relevant context, it is essential to study live suspension cell model systems especially since hematologic cancer models are inherently nonadherent.

Majority of the traditional techniques for measuring on-cell binding require labeling, bulk averaging, or strong immobilization, which can distort membranes, and obscure real-time kinetics.^11^ Moreover, many of the conventional techniques lack sensitivity or high-resolution to analyze interactions at the subcellular level, thereby missing key insights on binding heterogeneity.^12^ And while previous studies have investigated CA IX inhibitor binding in live cell environments, such approaches are fluorescent based, thus lacking direct detection and accurate quantification of the kinetic interactions.^13–15^

Surface Plasmon Resonance Microscopy (SPRM) overcomes these difficulties by enabling label-free, real-time measurement of molecular interactions on intact whole cells, thereby maintaining the receptor conformation and cellular microenvironment to deliver highly relevant physiological data.^16,17^ Additionally, SPRM’s single-cell spatial resolution allows for detailed assessment of cell-to-cell heterogeneity, making it especially well-suited for studying membrane-associated targets in smaller cells such as hematopoietic cancer cell.^18,19^

SPRM represents a paradigm shift in SPR-based applications, which excels in studying binding induced dielectric changes within heterogeneous structures, such as cells. It is typically implemented for kinetic binding measurements of protein interactions in fixed adherent cells. SPRM studies of live suspension cells have been avoided as they pose an extra measurement challenge over concerns of attachment instability and live micromotion behavior.^16,20,21^ However, as a pioneering first effort, SPRM is being implemented here to demonstrate the feasibility of kinetic binding interactions of small molecules on live suspension cells, drastically broadening the range of applications for this highly sensitive technique.

The best-studied class of CA IX inhibitors are sulfonamides due to their known mode of action, which involves the binding to the zinc ion within the enzyme’s catalytic site.^22,23^ This inhibitor class has been rationally redesigned through molecular engineering to increase tumor-specific isoform selectivity and affinity, such as for CA IX.^24^ The versatility of sulfonamides, and the extensive history of their therapeutic use, render them highly suitable for cancer therapy and targeted drug design.^25^ Furthermore, their ability to interfere with tumor pH homeostasis holds the promise of synergy between their use and existing treatments for lymphoma. Given the important role of sulfonamide inhibitors as strong and selective modulators of CA IX activity, characterizing their binding on live cells is essential for accurately predicting inhibitor performance under *in vivo* conditions.

Here, we pioneer a method using SPRM to study small molecule kinetic interactions label-free on live suspension cells and present the first in-depth characterization of five sulfonamide-based small molecule inhibitors (Acetazolamide, Sulfanilamide Furosemide, Dansylamide, and 4-Carboxybenzenesulfonamide) binding to CA IX on live Ramos B suspension cells. Furthermore, we compared the kinetic interactions of CA IX and CA II, which are cytosolic isoforms that share homologous active sites. Importantly, purified CA II is highly stable outside its native environment, uniquely making it extensively studied and well characterized for kinetic binding interactions using traditional techniques.^26,27^ By directly comparing *in vitro* native-membrane CA IX kinetic profiles with those of isolated *ex vitro* CA II, we reveal how membrane association modulates CA IX binding dynamics. Our findings offer a physiologically relevant model for probing CA IX activity in its native context and pioneer a strong foundation for the implementation of SPRM technology in studying label-free kinetic binding interactions of small molecules on whole live suspension cells.

## Experimental Section

### Materials and Reagents

A SPRm 200AP instrument and cell chamber kits (104–00228) from Biosensing Instrument Inc. were utilized. Poly L lysine BioReagent, suitable for cell culture (cat. # P8920) was purchased from Millipore Sigma. DMSO (Dimethyl sulfoxide catalog # J66650 AE) was purchased from Thermo Fisher Scientific. Small molecule inhibitors (Acetazolamide cat. # A6011, 4-carboxybenzenesulfonamide cat. # C11804, Furosemide cat. # 1287008, Sulfanilamide cat. # S9251 and Dansylamide cat. # 218898), each with a purity of 95% or higher as determined by HPLC were purchased from Millipore Sigma. Human CA IX Alexa Fluor 488-conjugated Antibody (catalog# FAB2188G-100UG), and Human CD20 (Research grade Rituximab Biosimilar) Alexa Fluor 488-conjugated antibody were purchased from R&D Systems. Alexa Fluor 488 antihuman TCR alpha/beta cat. # 306712 was purchased from Biolegend. Trypsin-EDTA solution, 1X (cat. # 30-2101) was purchased from ATCC. Dimethyl sulfoxide (DMSO) cat. # D4540 was purchased from Millipore Sigma.

### Cell Culture and Cell Seeding

Ramos B (RA1) cells (ATCC-CRL-1596) were cultured in Mccoy medium 5A (cat. # 16600082) supplemented with 10% FBS. All cells were maintained in an incubator at 37 °C with 5% CO_2_ before seeding. For every experiment, cell viability was determined with Trypan blue using a TC20 cell counter (BioRad). PLL precoated SPRM sensor chips were prepared according to manufacturer’s protocol (Gibco, cat. # A3890401). 5K Ramos B cells at 99% viability were gently seeded onto PLL precoated SPRM sensor chips containing 600 μL of Mccoy medium 5A supplemented with 10% FBS and then incubated at 37 °C with 5% CO_2_. After 72 h of incubation, cells were washed with live cell imaging buffer (A59688DJ) twice and immediately followed by SPRM analysis. This study did not involve human participants or animal subjects. All experiments were performed with a commercially available Ramos B cell line. As such, institutional review board approval was not required in accordance with ACS ethical guidelines.

### Surface Plasmon Resonance Microscopy

The SPRm 200AP (surface plasmon resonance microscope model 200AP from Biosensing Instrument Inc., Tempe, Arizona), which has an integrated microscope for simultaneous bright field imaging and SPR microscopy (600 μm × 450 μm viewing area), was used in this study. SPRM is highly sensitive to ligand binding events on the cell membrane, which induces dielectric changes that are recorded as sensorgrams. Moreover, its high-resolution diffraction limited optics produces sensorgrams of subcellular response regions, enabling detailed evaluation of binding heterogeneity.

The running buffer was a live cell imaging buffer with 0.05% DMSO and flown at a rate of 100 μL/min. The sensor chip containing live Ramos B cells was then exposed to a kinetic titration series of injections for each of the small molecule compounds (Acetazolamide, Furosemide, Dansylamide, 4-CBS, and Sulfanilamide) at 1/3 dilution for eight points from 40 μM (0.01, 0.05, 0.16, 0.4, 1.4, 4.4, 13.3, 40 μM) with a delay rinse in between each compound to allow for the complete dissociation of any bound compounds. The compound series was repeated three times in a random order.

### Fluorescence Microcopy

Preliminary conformation studies on evaluating cell type and receptor expression levels were performed by using fluorescence microscopy. Cells were seeded at 5 000 cells/ml in sensor chips. After culturing for 72 h at 37 °C and 5% CO_2_ in the media, cells were then incubated in blocking buffer for 1 h at 4 °C. Alexa fluor 488 labeled anti-CA IX, antihuman T cell receptor (TCR) and antihuman CD20 antibodies at 25 nM were each incubated for 15 min with cells and then imaged with fluorescence microscopy. ImageJ was used to analyze antibody fluorescence data.

### Data Fitting and Analysis

High resolution SPRM measurements were generated by uniformly segmenting the sensing area (600 μm × 450 μm area) into a virtual grid of 600 regions of interest (ROI). For every ROI, a 1:1 kinetic binding interaction model was applied. Bare area ROIs were subtracted as references from the cell area ROIs. The SPRM kinetic analysis results of all ROIs were aggregated for statistical analysis. ImageSPR software (Biosensing Instrument Inc., Tempe AZ) was used to generate the sensorgrams, fitting, and statistical analysis of binding interactions. ROIs that detected a response which fitted well to the kinetic interaction model were collectively plotted onto an isoaffinity scatter plot for statistical analysis of the binding heterogeneity (Figure 1). Histograms extracted from the isoaffinity scatter plot were fit with Gaussian distributions to obtain mean values and standard deviations for the association rate (*k*_a_), dissociation rate (*k*_d_), and equilibrium dissociation constant (KD). Subsequently, those ROIs that observe binding responses were highlighted in red and overlaid onto the SPRM image to more clearly evaluate the extent of cell-specific and nonspecific binding.

**Figure 1:**
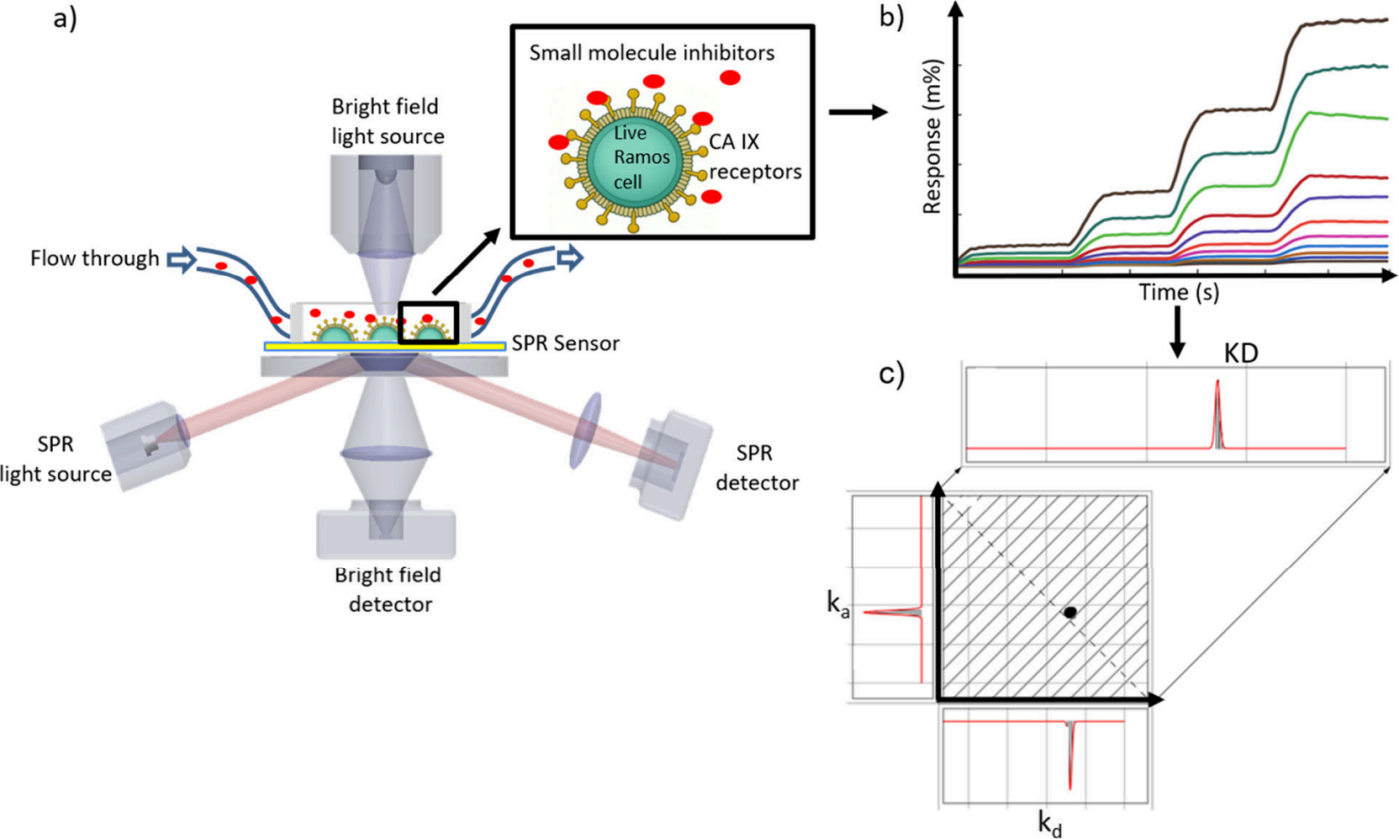
Measuring small molecule binding interactions on suspension cells using SPRM. a) Schematic of SPRM setup. SPRM light source induces SPR on sensor chip in the presence of Ramos B cells. Plasmonic resonance condition, which is extremely sensitive to changes in dielectric constant due to molecular binding, is recorded by SPRM detector. Simultaneous bright field (BF) imaging of detection area helps to convey cell morphology, confluency, and other phenotypes. Small molecules are delivered to the cells on the sensor surface via an automated microfluidics system though a flow cell, supporting simultaneous SPR and BF imaging. b) Entire SPR sensing area is uniformly divided into a virtual grid of 600 regions of interest (ROI). Simultaneous responses from every ROI during the kinetic titration injection series is recorded. Every RIO response series is fitted to 1:1 kinetic interaction model to extract kinetic parameters. c) The calculated ka and kd rates from every ROI’s binding interaction series are collected to form an isoaffinity scatter plot, displaying the heterogeneity of the binding interaction. Gaussian distributions are applied to the histograms along each axis and the diagonal to extract the mean and SD for the three kinetic parameters (*k*_a_, *k*_d_, and KD values).

## Results and Discussion

In this work, a new technique for measuring the binding kinetics and spatial distribution of small molecules interacting with surface receptors on intact live suspension cells was investigated using SPRM, enabling critical insights for synthetic chemistry, receptor biology, and advances in biophysical characterization.

### Cell Attachment and Receptor Confirmation

For this study of small molecule binding interactions with CA IX on the surface of live Ramos B cancer cells, the cells were grown on precoated PLL sensor chips and mounted on the SPRm 200 AP instrument as described in the methods section (see material and methods) (Figure 1). Among various commonly implemented cell-adhesion coatings tested in preliminary experiments, PLL provided the most consistent and stable adhesion behavior for Ramos B cells, showing minimal movement throughout the kinetic measurement assay. PLL is a positively charged polymer that promotes cell adhesion through electrostatic interactions with the negatively charged components of the cell membrane.^28–30^ An orthogonal detection method using fluorescence microscopy was implemented to verify the cell viability and surface-receptor accessibility (Figure 2). Fluorescence testing was implemented using probes specific to the CD-20, TCR, and CA IX receptors. Alexa fluor 488 labeled antibodies anti-CA IX, antihuman CD20, and as a negative control antihuman TCR at 25 nM were each incubated for 15 min with the cells and then imaged with fluorescence microscopy. Fluorescence results verified that expected levels of receptor expression continued after cell-seeding on the PLL surface, suggesting that the cells retained a healthy state while settled on the PLL surface.

**Figure 2:**
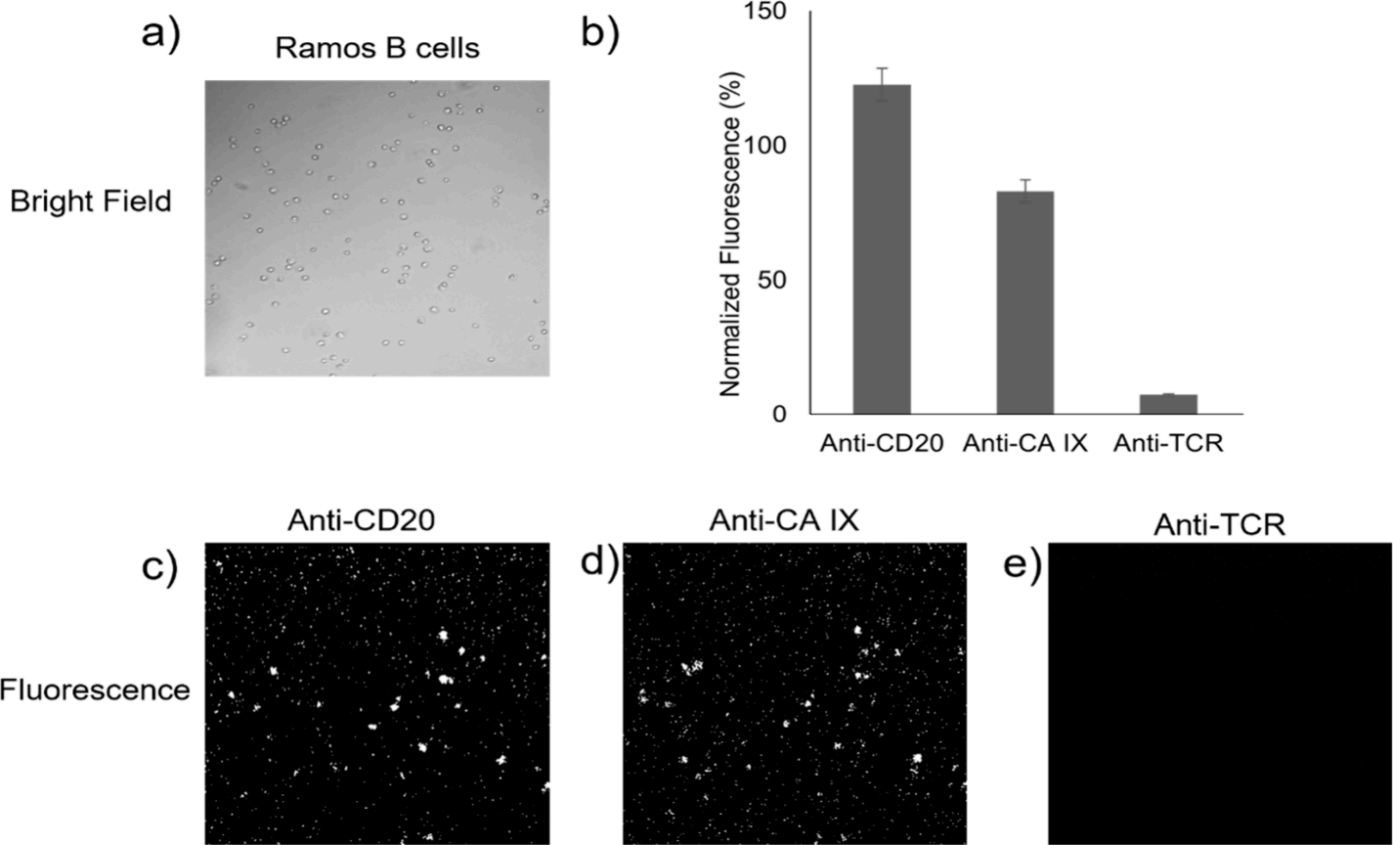
Fluorescence microscopy was performed to verify cell viability and receptor accessibly. a) Bright field image of Ramos B cells, b) Quantification through fluorescence analysis of the relative binding levels on Ramos B cells for c) anti-CD20 (Rituximab), and d) anti-CA IX, and e) anti-TCR negative control.

### Evaluating Live Cell Vitality

Bright field and SPR work together to confirm cell distribution, morphology, attachment, and vitality. In general, only viable live Ramos B cells remain attached to the sensor surface. Upon cell death, cells detach from the sensor surface, which is readily observed by their absence in the bright field image and single large-cell-specific jumps in the SPR baseline. Cell detachment was not observed during experimental runs, confirming the stable attachment of viable live Ramos B cells to the PLL coated sensor surface.

Additionally, the vitality of the live cells was monitored in real-time during each run by measuring the SPR baseline root-mean-square (RMS) noise values of each cell. Viable cells exhibit a substantially higher baseline RMS noise than fixed ones due to their micromotion, on average ∼5.4 and ∼2.5 m%, respectively (Supplemental Figure 1). Despite this 2.2-fold increase in baseline RMS noise exhibited with live cells, the average cell response due to small molecule binding is at least 5 times greater, in the several tens to hundreds of m% reflectivity range. Consequently, PLL proved to be an ideal surface for stabilizing Ramos B cells in their native physiological state while performing SPRM-based analysis.

### Cell-Based Analysis of Sulfonamides Binding to Live Suspension Cells

Five sulfonamide-based inhibitors (Acetazolamide, Sulfanilamide Furosemide, Dansylamide, and 4-CBS), which target the extracellular region of the CA IX receptor, were selected for this binding interaction study, as they have been well characterized in binding studies with other isoforms using traditional approaches.^31,32^ After performing a kinetic titration injection series of these small molecules on live Ramos B cells (see methods), data was analyzed as described in the data analysis section. Briefly, for each of the five compounds tested, high resolution analysis occurred with responses from 600 ROIs being fit to a 1:1 kinetic binding model (Supplemental Figure 2). The extracted kinetic parameters were then plotted in an isoaffinity scatter plot of association (*k*_a_) versus dissociation (*k*_d_) rate constants (Figure 3, Iso affinity). Histograms extracted from each isoaffinity scatter plot were fit with Gaussian distributions along the *y*-axis, *x*-axis, and diagonal to obtain the mean and standard deviation for the *k*_a_, *k*_d_, and KD values, respectively. For each of the five compounds, the binding interaction experiment was repeated thrice. Isoaffinity scatter plots for each compound revealed well-clustered populations of binding events. Histograms for KD displayed sharp peaks that fit well with the Gaussian distribution, providing mean KD values and 95% confidence intervals (Figure 3, KD Distribution). Additionally, those ROIs that observed binding were highlighted in red and overlaid on the SPRM image as an activity map to facilitate evaluation of cell-specific and nonspecific binding activity (Figure 3, SPR image). Negligible binding responses were observed from bare areas, indicating high cell specificity for all compounds. Results of the kinetic interaction analysis for all five compounds are summarized in Table 1.

**Figure 3:**
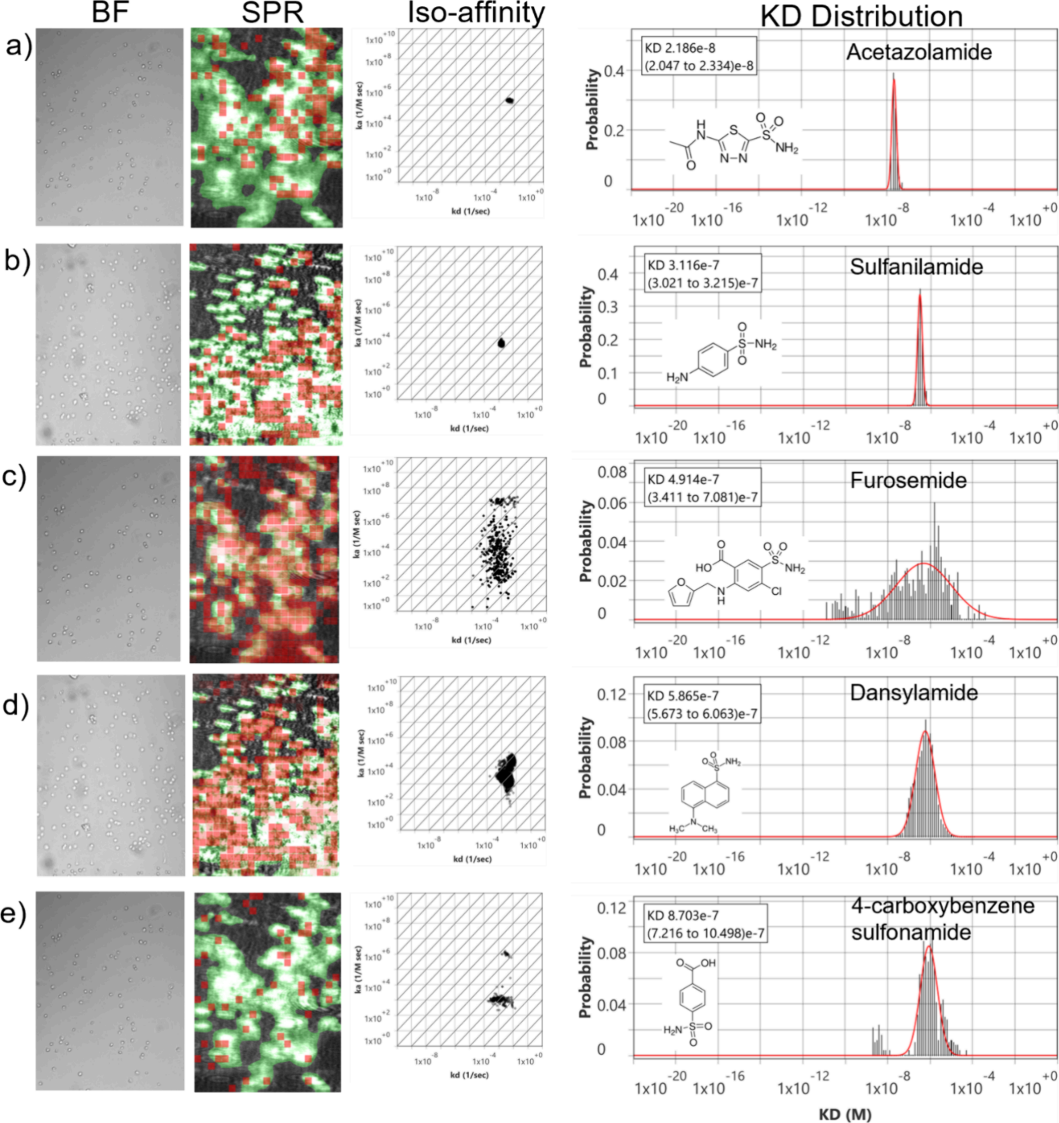
Small molecule binding kinetics on Live Ramos B cell surface. Bright field image of Ramos B cells on the sensor surface and its corresponding SPR image. Red squares are regions of interest (ROIs) that observe responses which closely fit the kinetic binding model. The green regions indicate areas confluent with cells. Active areas designated by red ROIs overlap closely with cell regions indicating high cell specificity. The measured interactions of a) Acetazolamide, b) Sulfanilamide, c) Furosemide, d) Dansylamide, and e) 4-CBS with CA IX receptors on the surface of live Ramos B cells are presented in isoaffinity scatter plots to reveal binding heterogeneity and predominant modes of interaction. The KD histograms extracted from each isoaffinity scatter plot were fitted with Gaussian distribution to statistically extract the mean and distribution of the kinetic parameter.

**Table 1:** Kinetics of Small Molecule Binding Interactions to *In Vitro* Membrane-Bound CA IX and *Ex Vitro* Purified CA II Influence of Live Native Membrane on CA IX Binding Interaction

	SPRM results for CA IX			
Small molecules	KD (nM)	*k*_a_ (M^–1^ s^–1^)	*k_d_* (s^–1^)	Reported values CA II^40^
Acetazolamide	21	2.08 × 10^05^	4.68 × 10^–03^	19 nM
Sulfanilamide	311	4.40 × 10^03^	1.39 × 10^–03^	5 μM
Furosemide	491	1.10 × 10^03^	1.16 × 10^–03^	513 nM
Dansylamide	586	2.53 × 10^03^	2.34 × 10^–03^	760 nM
4-CBS	870	1.10 × 10^03^	1.19 × 10^–03^	893 nM

In studying the binding interactions of a complex and heterogeneous environment such as that of the cell surface, it is not readily possible to unambiguously confirm the extent of binding specificity to the targeted receptor site. However, several key considerations strongly suggest that the observed cellular responses due to binding are likely specific to or predominantly related to the CA IX target. The best-studied class of CA inhibitors are sulfonamides due to their known mode of action.^22,23^ Admittedly, sulfonamide derivatives are known to exhibit selective binding toward other types of receptors, of which the most significant secondary targets include carbonic anhydrases, cyclooxygenases, and ATP-sensitive potassium channel subunits. Fortunately, in this case with Ramos B cells, CA IX is the only receptor reported to be expressed extracellularly at detectable levels, thereby making CA IX the most likely contributor to the SPR binding signal..^7,33^ Furthermore, in the case of 4-CBS, it is highly preferential to the CA IX isoform over all other isoforms. Similarly, the other compounds tested in this study produced binding responses at levels similar to that of 4-CBS, though at varying kinetic rates, supporting a high likelihood that their interactions are also with the CA IX receptor.^31^ With these arguments taken together, we attribute the SPRM binding responses to be predominantly from selective interaction with the extracellularly located and overexpressed CA IX receptor.

### Comparison of Structure–Affinity Relationships of Small Molecule Interactions

The catalytic site of CA IX varies in its structure and amino acid composition especially surrounding the catalytic zinc ion, providing varied binding affinities to different inhibitors.^34,35^ The catalytic site of CA IX has a catalytic zinc ion bound to three histidine residues (His94, His96, and His119) and a water molecule or hydroxide ion, forming a tetrahedral shape.^23,36^ The sulfonamide group present in the small molecule inhibitors is a zinc-binding group that quickly replaces the bound water molecule and binds the zinc ion. This aspect is critical for effective target inhibition.^37,38^

Acetazolamide, a clinically established carbonic anhydrase inhibitor, exhibited a KD of 21 nM, it exhibited the highest affinity among the five compounds with the CA IX receptor, corroborating previously reported values (Figure 3a).^39,40^ The association rate constant (*k*_a_ = 2.08 × 10^05^ M^–1^ s^–1^) was markedly higher than the other inhibitors, indicating a rapid and tight engagement of the enzyme’s active site. The compact nature of the compound and optimal zinc binding geometry most likely account for its improved kinetics and equilibrium binding affinity.^41–43^ The deprotonated sulfonamide nitrogen in acetazolamide directly interacts with Zn^2+^ in the binding site of the receptor. Moreover, the strong binding affinity in CA IX and isoforms results from this compound’s electrostatic complementarity to the enzyme’s active site, resulting in efficient molecular recognition without radical conformational changeses.^34,44^

Sulfanilamide, a classic sulfonamide scaffold, exhibited a KD of 311 nM, indicating moderate binding to CA IX (Figure 3b). Also, the aromatic groups present in sulfanilamide can interact with the well exposed Phe131 in the active site conferring higher binding affinity in CA IX.^45^ Furosemide, a sulfonamide derivative loop diuretic, exhibited a KD of 491 nM, (Figure 3c). Dansylamide, with its bulky aromatic naphthalene sulfonamide skeleton, showed a KD of 586 nM (Figure 3d). While the large hydrophobic area could allow favorable π–π interactions with the aromatic residues surrounding the CA IX pocket, the bulkiness of Dansylamide is anticipated to lead to some stereochemical constraints being placed on the optimal coordination geometry with the catalytic zinc ion. 4-CBS shows the weakest binding affinity for CA IX, with a KD of 870 nM (Figure 3e). Even though electron-withdrawing groups can form hydrogen bonds or electrostatic interactions with the nearby residues, the substitution pattern of the aromatic ring could disrupt the inhibitor’s ability to effectively orient the sulfonamide group toward the zinc ion present in the active site of the enzyme.

Purified CA IX is inherently insoluble and unstable outside its native membrane environment making it difficult to obtain kinetic values that accurately reflect its *in vivo* function.^46^ In a pioneering effort, SPRM is implemented here to measure CA IX interactions in live Ramos B cells, thereby protecting native attributes of the cellular environment, such as membrane context, glycosylation, and lipid content. In contrast, CA II, a well-characterized isoform with homologues binding site, is highly stable in its purified form.^26,31,40,47^ By comparing the published *ex vitro* CA II kinetic interaction results against SPRM’s *in vitro* CA IX interaction results, an evaluation of SPRM’s performance in this unique application can be achieved.

As can be seen from the comparison in Table 1, with the noteworthy exception of sulfanilamide, the compounds show similar binding kinetics with an overall match on rank order of the KD values between the two isoforms, validating the utility of SPRM.

Interestingly, sulfanilamide shows ∼16 times greater affinity for *in vitro* CA IX than for *ex vitro* CA II. While the catalytic domains of CA IX and CA II are largely homologous, the membrane-associated context of CA IX provides a more open polar pocket with favorable hydrogen bonding and electrostatic stabilization of the sulfanilamide which is absent when the receptor is extracted from its native membrane context.^34^ These findings strongly suggest that both the structural features of the active site and the membrane context may have worked together to enhance the binding of sulfanilamide to CA IX. This unique observation is not readily possible using traditional kinetic measurement methods, which require membrane disruption or labeling.

To further evaluate the accuracy of the SPRM assay in detecting small molecule interactions on live suspension cells, the interassay precision and reproducibility was determined. Each compound was tested in triplicate on separate days, sensor chips, and batches of independent cell cultures. The average interassay percent coefficient of variation (%CV) ranged from 4 to 10% across all five compounds (Table 2), and an overall low %CV of 6.8% was observed, reflecting high robustness and accuracy for SPRM in this pioneering application of measuring small molecule interactions on live suspension cells.

**Table 2:** A Total of 3 SPRM Experiments Were Performed on Small Molecule Inhibitors on Live Ramos B Cells from Different Batches and Different Sensor Chips^a^

	Batch					
Compounds	1	2	3	Mean	SD	%CV
Acetazolamide	21 nM	18 nM	20 nM	19	1	7
Sulfanilamide	311 nM	364 nM	317 nM	330	29	8
Furosemide	491 nM	523 nM	470 nM	494	21	4
Dansylamide	586 nM	542 nM	534 nM	554	28	5
4-CBS	870 nM	1 μM	780 nM	883	90	10
Overall						6.8

a KD values for each compound were pooled from across all 3 different experiments to determine the mean, standard deviation (SD), and coefficients of variation (%CV).

## Conclusion

CA IX receptor represents a high-profile target for drug development but remains elusive as it cannot be readily studied outside the native membrane environment using traditional kinetic analysis techniques. Here, we present a pioneering approach that implements SPRM to study CA IX interactions in live Ramos B suspension cells *in vitro* with five sulfonamide-based small molecule inhibitors. In comparison to binding studies that use traditional membrane-free *ex vitro* approaches with CA II, a readily purified isoform with a homologous active binding site, binding affinities have similar values and match overall rank order, except for Sulfanilamide. With Sulfanilamide, we observe here a unique ∼16 times increase in affinity for the native-form CA IX relative to the purified CA II isoform. We attribute this significant difference to an enhancing influence of the native membrane environment on active site topography and receptor function. Importantly, the noticeable increase in affinity suggests sulfanilamide may warrant renewed consideration as a lead scaffold for carbonic anhydrase-based drug development when evaluated under biologically relevant conditions. Additionally, we report a low 6.8% CV for SPRM in this pioneering application of measuring small molecule interactions on live suspension cells. Our findings validate SPRM for live suspension cell-based kinetic binding studies with small molecule compounds and highlight the importance of *in vitro* based assays, as they produce a more physiologically relevant evaluation of binding interactions to membrane-bound targets than traditional approaches.

## Supplementary Material



## Notes

RMS% graph to show baseline noise in live vs fixed cells and raw SPRM sensograms (PDF)

## Abbreviations

CA IX: Carbonic anhydrase IX
CA II: Carbonic anhydrase II
4-CBS: 4-Carboxybenzene sulfonamide
SPRM: Surface plasmon resonance microscopy
TCR: T-cell receptor
CV: coefficient of variation
RMS: root-mean-square

## References

[1] Wykoff C. C., Beasley N. J. P., Watson P. H., Turner K. J., Pastorek J., Sibtain A., Wilson G. D., Turley H., Talks K. L., Maxwell P. H., Pugh C. W., Ratcliffe P. J., Harris A. L. (2000). Hypoxia-Inducible Expression of Tumor-Associated Carbonic
Anhydrases. Cancer Res..

[2] Pastorekova S., Gillies R. J. (2019). The Role of Carbonic
Anhydrase IX in Cancer Development:
Links to Hypoxia, Acidosis, and Beyond. Cancer
and Metastasis Reviews..

[3] McDonald P. C., Winum J. Y., Supuran C. T., Dedhar S. (2012). Recent Developments
in Targeting Carbonic Anhydrase IX for Cancer Therapeutics. Oncotarget.

[4] Becker H. M. (2020). Carbonic
Anhydrase IX and Acid Transport in Cancer. British
Journal of Cancer..

[5] Nasu K., Yamaguchi K., Takanashi Y., Sugamura K., Harigae H. (2015). Carbonic Anhydrase
IX Promotes the Tumorigenicity of Adult T-Cell Leukemia/Lymphoma-Derived
Cells in NOG Mice. Blood.

[6] Nasu K., Yamaguchi K., Takanashi T., Tamai K., Sato I., Ine S., Sasaki O., Satoh K., Tanaka N., Tanaka Y., Fukushima T., Harigae H., Sugamura K. (2017). Crucial Role of Carbonic
Anhydrase IX in Tumorigenicity of Xenotransplanted Adult T-Cell Leukemia-Derived
Cells. Cancer Sci..

[7] Chen L. Q., Howison C. M., Spier C., Stopeck A. T., Malm S. W., Pagel M. D., Baker A. F. (2015). Assessment
of Carbonic Anhydrase
IX Expression and Extracellular PH in B-Cell Lymphoma Cell Line Models. Leuk Lymphoma.

[8] McDonald P. C., Chafe S. C., Supuran C. T., Dedhar S. (2022). Cancer Therapeutic
Targeting of Hypoxia Induced Carbonic Anhydrase IX: From Bench to
Bedside. Cancers.

[9] Koruza K., Murray A. B., Mahon B. P., Hopkins J. B., Knecht W., McKenna R., Fisher S. Z. (2020). Biophysical Characterization of Cancer-Related
Carbonic Anhydrase Ix. Int. J. Mol. Sci..

[10] Askoxylakis V., Garcia-Boy R., Rana S., Krämer S., Hebling U., Mier W., Altmann A., Markert A., Debus J., Haberkorn U. (2010). A New Peptide
Ligand for Targeting
Human Carbonic Anhydrase IX, Identified through the Phage Display
Technology. PLoS One.

[11] Hunter, S. A. ; Cochran, J. R. Cell-Binding Assays for Determining the Affinity of Protein–Protein Interactions: Technologies and Considerations. In Methods Enzymol. 2016, 580. 10.1016/bs.mie.2016.05.002. PMC6067677 27586327

[12] Weber, J. ; Djurberg, K. ; Lundsten Salomonsson, S. ; Kamprath, M. ; Hoehne, A. ; Westin, H. ; Vergara, F. ; Bondza, S. Modelling Ligand Depletion for Simultaneous Affinity and Binding Site Quantification on Cells and Tissue. Sci. Rep 2023, 13 (1). 10.1038/s41598-023-37015-1. PMC10282064 37340068

[13] Matuliene, J. ; Zvinys, G. ; Petrauskas, V. ; Kvietkauskaite, A. ; Zaksauskas, A. ; Shubin, K. ; Zubriene, A. ; Baranauskiene, L. ; Kacenauskaite, L. ; Kopanchuk, S. ; Veiksina, S. ; Paketuryte-Latve, V. ; Smirnoviene, J. ; Juozapaitiene, V. ; Mickeviciute, A. ; Michailoviene, V. ; Jachno, J. ; Stravinskiene, D. ; Sliziene, A. ; Petrosiute, A. ; Becker, H. M. ; Kazokaite-Adomaitiene, J. ; Yaromina, A. ; Capkauskaite, E. ; Rinken, A. ; Dudutiene, V. ; Dubois, L. J ; Matulis, D. Picomolar Fluorescent Probes for Compound Affinity Determination to Carbonic Anhydrase IX Expressed in Live Cancer Cells. Sci. Rep. 2022, 12 (1). 10.1038/s41598-022-22436-1. PMC9586938 36271018

[14] Friedman Ohana, R. ; Hurst, R. ; Rosenblatt, M. ; Levin, S. ; Machleidt, T. ; Kirkland, T. A. ; Encell, L. P. ; Robers, M. B. ; Wood, K. V. Utilizing a Simple Method for Stoichiometric Protein Labeling to Quantify Antibody Blockade. Sci. Rep. 2019, 9 (1). 10.1038/s41598-019-43469-z. PMC6504924 31065015

[15] Dietz M. S., Wehrheim S. S., Harwardt M. L. I. E., Niemann H. H., Heilemann M. (2019). Competitive
Binding Study Revealing the Influence of Fluorophore Labels on Biomolecular
Interactions. Nano Lett..

[16] Zhou X. L., Yang Y., Wang S., Liu X. W. (2020). Surface Plasmon
Resonance Microscopy: From Single-Molecule Sensing to Single-Cell
Imaging. Angewandte Chemie - International Edition..

[17] Mamer S. B., Page P., Murphy M., Wang J., Gallerne P., Ansari A., Imoukhuede P. I. (2020). The Convergence
of Cell-Based Surface
Plasmon Resonance and Biomaterials: The Future of Quantifying Bio-Molecular
Interactions—A Review. Ann. Biomed Eng..

[18] Bocková M., Slabý J., Špringer T., Homola J. (2019). Advances in Surface
Plasmon Resonance Imaging and Microscopy and Their Biological Applications. Annual Review of Analytical Chemistry..

[19] Bocková M., Slabý J., Špringer T., Homola J. (2019). Annual Review of Analytical
Chemistry Advances in Surface Plasmon Resonance Imaging and Microscopy
and Their Biological Applications. Annual Review
of Analytical Chemistry.

[20] Wang W., Yang Y., Wang S., Nagaraj V. J., Liu Q., Wu J., Tao N. (2012). Label-Free Measuring and Mapping of Binding Kinetics
of Membrane Proteins in Single Living Cells. Nat. Chem..

[21] Zhang F., Jing W., Hunt A., Yu H., Yang Y., Wang S., Chen H. Y., Tao N. (2018). Label-Free
Quantification
of Small-Molecule Binding to Membrane Proteins on Single Cells by
Tracking Nanometer-Scale Cellular Membrane Deformation. ACS Nano.

[22] Wingo T., Tu C., Laipis P. J., Silverman D. N. (2001). The Catalytic Properties of Human
Carbonic Anhydrase IX. Biochem. Biophys. Res.
Commun..

[23] Tu C., Foster L., Alvarado A., McKenna R., Silverman D. N., Frost S. C. (2012). Role of Zinc in Catalytic Activity of Carbonic Anhydrase
IX. Arch. Biochem. Biophys..

[24] Supuran C. T. (2012). Inhibition
of Carbonic Anhydrase IX as a Novel Anticancer Mechanism. World J. Clin Oncol.

[25] Elsayad K. A., Elmasry G. F., Mahmoud S. T., Awadallah F. M. (2024). Sulfonamides
as Anticancer Agents: A Brief Review on Sulfonamide Derivatives as
Inhibitors of Various Proteins Overexpressed in Cancer. Bioorg Chem..

[26] Day Y. S. N., Baird C. L., Rich R. L., Myszka D. G. (2002). Direct Comparison
of Binding Equilibrium, Thermodynamic, and Rate Constants Determined
by Surface- and Solution-based Biophysical Methods. Protein Sci..

[27] Di
Fiore A., Alterio V., Monti S. M., De Simone G., D’Ambrosio K. (2015). Thermostable Carbonic Anhydrases in Biotechnological
Applications. International Journal of Molecular
Sciences..

[28] Mazia D., Schatten G., Sale W. (1975). Adhesion of
Cells to Surfaces Coated
with Polylysine: Applications to Electron Microscopy. J. Cell Biol..

[29] Rodig S. J. (2020). Attaching
Suspension Cells to Slides for Staining. Cold
Spring Harb Protoc.

[30] Hategan A., Sengupta K., Kahn S., Sackmann E., Discher D. E. (2004). Topographical
Pattern Dynamics in Passive Adhesion of Cell Membranes. Biophys. J..

[31] Cannon M. J., Papalia G. A., Navratilova I., Fisher R. J., Roberts L. R., Worthy K. M., Stephen A. G., Marchesini G. R., Collins E. J., Casper D., Qiu H., Satpaev D., Liparoto S. F., Rice D. A., Gorshkova I. I., Darling R. J., Bennett D. B., Sekar M., Hommema E., Liang A. M., Day E. S., Inman J., Karlicek S. M., Ullrich S. J., Hodges D., Chu T., Sullivan E., Simpson J., Rafique A., Luginbühl B., Westin S. N., Bynum M., Cachia P., Li Y. J., Kao D., Neurauter A., Wong M., Swanson M., Myszka D. G. (2004). Comparative
Analyses of a Small Molecule/Enzyme Interaction by Multiple Users
of Biacore Technology. Anal. Biochem..

[32] Nair S. K., Elbaum D., Christianson D. W. (1996). Unexpected
Binding Mode of the Sulfonamide
Fluorophore 5-Dimethylamino-1-Naphthalene Sulfonamide to Human Carbonic
Anhydrase II Implications for the Development of a Zinc Biosensor. J. Biol. Chem..

[33] Kardosh A., Wang W., Uddin J., Petasis N. A., Hofman F. M., Chen T. C., Schönthal A. H. (2005). Dimethyl-Celecoxib (DMC), a Derivative
of Celecoxib That Lacks Cyclooxygenase-2-Inhibitory Function, Potently
Mimics the Anti-Tumor Effects of Celecoxib on Burkitt’s Lymphoma
in Vitro and in Vivo. Cancer Biol. Ther.

[34] Moeker J., Mahon B. P., Bornaghi L. F., Vullo D., Supuran C. T., McKenna R., Poulsen S. A. (2014). Structural
Insights into Carbonic
Anhydrase IX Isoform Specificity of Carbohydrate-Based Sulfamates. J. Med. Chem..

[35] Dudutiene V., Matuliene J., Smirnov A., Timm D. D., Zubriene A., Baranauskiene L., Morkunaite V., Smirnoviene J., Michailoviene V., Juozapaitiene V., Mickevičiute A., Kazokaite J., Bakšyte S., Kasiliauskaite A., Jachno J., Revuckiene J., Kišonaite M., Pilipuityte V., Ivanauskaite E., Milinavičiute G., Smirnovas V., Petrikaite V., Kairys V., Petrauskas V., Norvaišas P., Linge D., Gibieža P., Čapkauskaite E., Zakšauskas A., Kazlauskas E., Manakova E., Gražulis S., Ladbury J. E., Matulis D. (2014). Discovery
and Characterization of Novel Selective Inhibitors of Carbonic Anhydrase
IX. J. Med. Chem..

[36] Rai D., Khatua S., Taraphder S. (2022). Structure
and Dynamics of the Isozymes
II and IX of Human Carbonic Anhydrase. ACS Omega.

[37] Pinard M. A., Mahon B., McKenna R. (2015). Probing the
Surface of Human Carbonic
Anhydrase for Clues towards the Design of Isoform Specific Inhibitors. BioMed. Research International..

[38] Alterio V., Hilvo M., Di Fiore A., Supuran C. T., Pan P., Parkkila S., Scaloni A., Pastorek J., Pastorekova S., Pedone C., Scozzafava A., Monti S. M., De Simone G. (2009). Crystal Structure
of the Catalytic Domain of the Tumor-Associated Human Carbonic Anhydrase
IX. Proc. Natl. Acad. Sci. U. S. A..

[39] Huwaimel B. I., Jonnalagadda S. K., Jonnalagadda S., Kumari S., Nocentini A., Supuran C. T., Trippier P. C. (2023). Selective Carbonic Anhydrase IX and
XII Inhibitors Based around a Functionalized Coumarin Scaffold. Drug Dev Res..

[40] Myszka D. G. (2004). Analysis
of Small-Molecule Interactions Using Biacore S51 Technology. Anal. Biochem..

[41] Supuran C. T. (2008). Carbonic
Anhydrases: Novel Therapeutic Applications for Inhibitors and Activators. Nature Reviews Drug Discovery..

[42] Supuran C. T. (2011). Carbonic
Anhydrase Inhibitors and Activators for Novel Therapeutic Applications. Future Medicinal Chemistry..

[43] Supuran C. T. (2018). Carbonic
Anhydrase Inhibitors as Emerging Agents for the Treatment and Imaging
of Hypoxic Tumors. Expert Opinion on Investigational
Drugs..

[44] Andring J. T., Fouch M., Akocak S., Angeli A., Supuran C. T., Ilies M. A., McKenna R. (2020). Structural
Basis of Nanomolar Inhibition
of Tumor-Associated Carbonic Anhydrase IX: X-Ray Crystallographic
and Inhibition Study of Lipophilic Inhibitors with Acetazolamide Backbone. J. Med. Chem..

[45] Menchise V., De Simone G., Alterio V., Di Fiore A., Pedone C., Scozzafava A., Supuran C. T. (2005). Carbonic Anhydrase
Inhibitors: Stacking
with Phe131 Determines Active Site Binding Region of Inhibitors as
Exemplified by the X-Ray Crystal Structure of a Membrane-Impermeant
Antitumor Sulfonamide Complexed with Isozyme II. J. Med. Chem..

[46] Hilvo M., Baranauskiene L., Salzano A. M., Scaloni A., Matulis D., Innocenti A., Scozzafava A., Monti S. M., Di Fiore A., De Simone G., Lindfors M., Jänis J., Valjakka J., Pastoreková S., Pastorek J., Kulomaa M. S., Nordlund H. R., Supuran C. T., Parkkila S. (2008). Biochemical Characterization
of CA IX, One of the Most Active Carbonic Anhydrase Isozymes. J. Biol. Chem..

[47] Rich R. L., Myszka D. G. (2007). Higher-Throughput, Label-Free, Real-Time Molecular
Interaction Analysis. Anal. Biochem..

