# Supplementary material for: Real-Time Binding Kinetics of Small Molecules to CA IX in Live Suspension Cells Using SPR Microscopy
Source: ACS Med Chem Lett. 2025 Dec 20;17(1):154–61. doi: 10.1021/acsmedchemlett.5c00555 (PMC12794078; doi:10.1021/acsmedchemlett.5c00555)
Supplement: Supplementary file 1 [file ml5c00555_si_001.pdf]

Real-Time Binding Kinetics of Small Molecules to CA IX in Live Suspension Cells Using SPR Microscopy

Miyuki A Thirumurthy[a], Jesús Aguilar Díaz de león[a], Nguyen Ly [a*]

[a]Biosensing Instrument Inc., Tempe, Az, USA

[*]Corresponding Author Email: Win@BiosensingInstrument.com.
Biosensing Instrument Inc., Tempe, Arizona, USA. Phone: 480-491-2777.

Supplemental Figure 1:

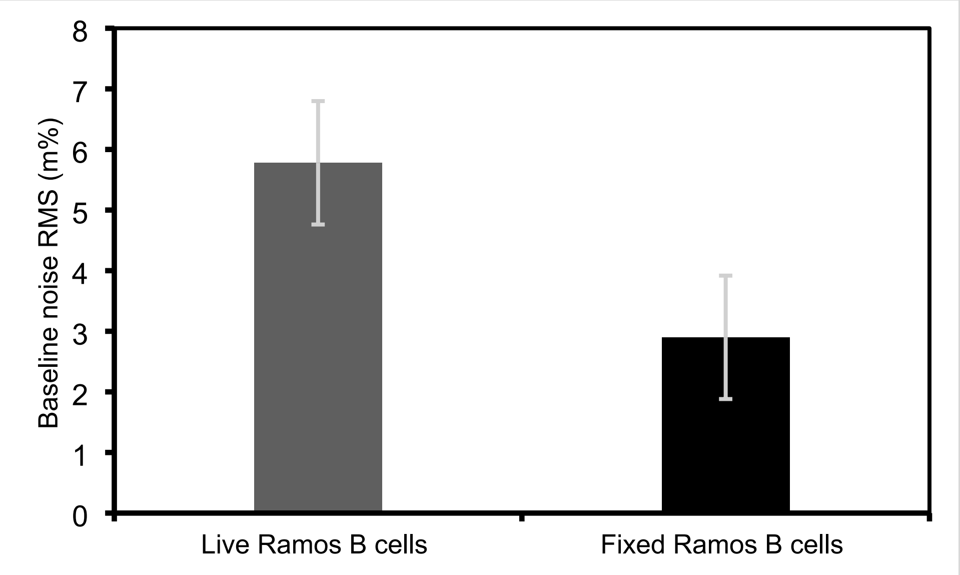

Supplemental Figure 1: Evaluation of cell vitality. *Comparison of baseline root mean square (RMS) fluctuations between live and fixed Ramos B cells. Live cells exhibited significantly higher RMS values (mean = 5.4 ± 0.5 m%) compared to fixed cells (mean = 2.5 ± 0.6 m%), which is attributed to live-cell micromotion.*

Supplemental Figure 2:

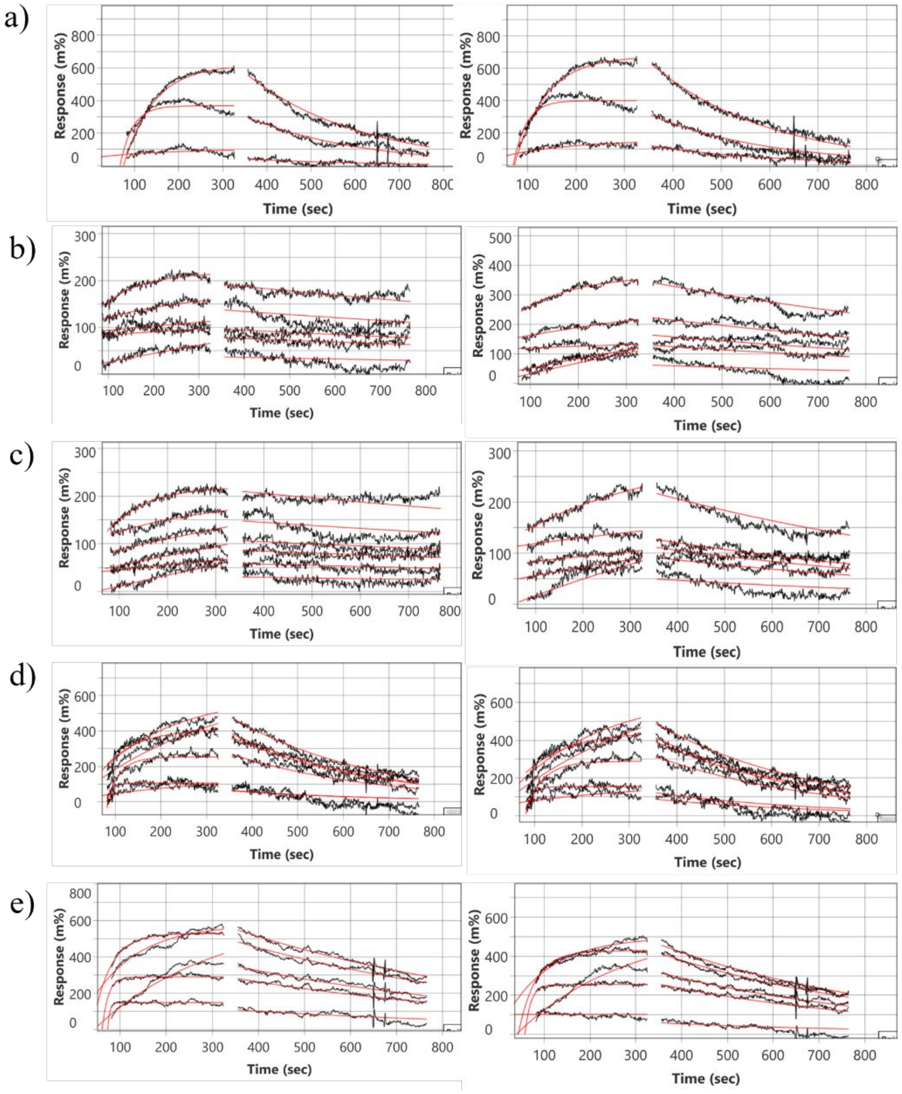

Supplemental Figure 2: *Sample of SPRM sensorgrams showing real-time binding kinetics of a) Acetazolamide, b) Sulfanilamide, c) Furosemide, d) Dansylamide, and e) 4-CBS with CA IX receptors on the surface of live Ramos B cell. Fits from 1:1 kinetic interaction model (red) overlay the data (black).*
